# Early coordinated multidisciplinary intervention to prevent sickness absence and labour market exclusion in patients with low back pain: study protocol of a randomized controlled trial

**DOI:** 10.1186/1471-2474-14-93

**Published:** 2013-03-13

**Authors:** Annette Fisker, Henning Langberg, Tom Petersen, Ole Steen Mortensen

**Affiliations:** 1 Department of Public Health, CopenRehab, University of Copenhagen, Øster Farimagsgade 5, DK-1014, Copenhagen K, Denmark; 2 Back- and Rehabilitation Centre of Copenhagen, Hans Knudsens Plads 3D, DK-2100, Copenhagen O, Denmark; 3 National Research Centre for the Working Environment, Lersø Parkallé 105, DK-2100, Copenhagen O, Denmark; 4 Department of Occupational Health, Køge Hospital, Lykkebækvej 1, DK-4600, Køge, Denmark

**Keywords:** Low back pain (LBP), Return to work (RTW), Sickness absence, Rehabilitation, Prevention, Multidisciplinary intervention, Coordination, Denmark

## Abstract

**Background:**

Musculoskeletal disorders account for one third of the long-term absenteeism in Denmark and the number of individuals sick listed for more than four weeks is increasing. Compared to other diagnoses, patients with musculoskeletal diseases, including low back pain, are less likely to return to work after a period of sick leave. It seems that a multidisciplinary intervention, including cooperation between the health sector, the social sector and in the work place, has a positive effect on days off work due to musculoskeletal disorders and particularly low back pain. It is a challenge to coordinate this type of intervention, and the implementation of a return-to-work (RTW)-coordinator is suggested as an effective strategy in this process. The purpose of this paper is to describe the study protocol and present a new type of intervention, where the physiotherapist both has the role as RTW-coordinator and treating the patient.

**Methods/design:**

A randomized controlled trial (RCT) is currently on-going. The RCT includes 770 patients with low back pain of minimum four weeks who are referred to an outpatient back centre. The study population consists of patients, who are sick-listed or at risk of sick-leave due to LBP. The control group is treated with usual care in a team of a physiotherapist, a chiropractor, a rheumatologist and a social worker employed at the centre. The Intervention group is treated with usual care and in addition intervention of a psychologist, an occupational physician, an ergonomist, a case manager from the municipal sickness benefit office, who has the authority in the actual case concerning sickness benefit payment and contact to the patients employer/work place. The treating physiotherapist is the RTW-coordinator. Outcome will be reported at the end of treatment as well as 6 and 12 months follow up. The primary outcome is number of days off work. Secondary outcomes are disability, pain, and quality of life. The study will follow the recommendations in CONSORT-statement in designing and reporting RCTs.

**Discussion:**

This large RCT is testing the effectiveness of a preventive intervention targeting patients on short term sick leave or at risk being sick listed because of low back pain. We have developed a novel multidisciplinary team structure using the treating physiotherapist as the return to work coordinator, and having the case manager from the municipal sickness benefit office participating in team meetings. The study has the potential to contribute to the knowledge about how to target the challenges in the treatment of LBP. The aim is to prevent sickness absence and labour market exclusion - both on the individual level and economic costs at community level. Short term results will be available in 2014.

This study is approved by the Danish Regional Ethics Committee (J.nr: H-C-2008-112) and is registered at.

**Trial registration:**

ClinicalTrials.gov: NCT01690234

## Background

Musculoskeletal diseases are some of the most prevalent health problems in the western societies. In particular low back pain (LBP) causes substantial disability among working age adults and are costly for society in both expenses due to health services, sickness absence and job loss [1].

The increase of sickness absence has been a concern in Denmark and most other European countries for some time [2]. In average 5% of the Danish workforce was sick listed in 2008 [3]. Musculoskeletal diseases cause more than 30% of the sickness absence in Denmark and back disorders alone cause 17% of the total long term sickness absence. Annual public expenditure to back related disorders is estimated to be 2.3 billion euro [4], mostly as expenditures for sickness absence benefit and early retirement. Promoting labour market participation is essential for Denmark and other European countries that face a decline in the proportion of residents in the working age due to an aging work force [5].

### Predictors

Several different predictors for long term sickness absence due to LBP are suggested [6-12], but the following have earlier been the most consistently reported: age above 50 years, high level of pain intensity, female gender [6,7], high level on self-reported disability scores [6,8], sciatic pain [6,7], social or psychological problems [6,7,9], low expectations on return to work [10], fear avoidance beliefs [7,8,10], low job satisfaction [7,11,12], recurrent episodes of sickness absence [7,12], unemployment [8,10] and economic gain (i.e. retirement) [6,7,9]. In addition long duration of sickness absence seems to be a predictor for labour market exclusion in LBP patients [7,10]. The variation of risk factors and their possible interaction underpins, that sickness absence due to LBP is a complex problem, and an optimal rehabilitation should be tailored to target all of the issues that are modifiable. Rehabilitation is in this study understood as a bio-psycho-social conceptual framework as in the definition based on WHOs ICF-terms [13,14].

### Recommendations

Due to the high recurrence rate of LBP in the adult population and the lack of consistency in the knowledge of predictors for the first episode of LBP the recommendations from The European Commission Research Directorate General, Backpain Europe [15], are to focus on preventing future consequences of LBP, e.g. reduced work ability, sickness absence and labour market exclusion.

The guidelines from The British National Institute of Clinical Excellence, NICE, on long term sickness absence recommend an early, multidisciplinary intervention, planned in cooperation with the workplace [16]. To facilitate the return to work (RTW) process, the Institute for Work and Health in Toronto, Canada recommends multidisciplinary interventions with an active involvement of a designated RTW coordinator as a key element in the process. The RTW coordinator should be responsible of identifying barriers to RTW, keeping the RTW plan on track and obtain support from healthcare providers and employers [17]. However, there is no information on which professional qualifications the RTW-coordinator must have.

A recent Danish Health Technology Assessment found moderate evidence that a coordinated multidisciplinary intervention is more effective at a clinically relevant level than mono disciplinary interventions on reducing sickness absence due to long-term LBP[18]. A review made to clarify the most important factors in the RTW process in persons sick listed as a result of musculoskeletal conditions concludes, that job modifications based on a work place visit, is recommended to facilitate the RTW process [2].

Physical exercise with special attention to fear avoidance is incorporated in most multidisciplinary interventions [19], and cognitive behavioural therapy (CBT) have earlier indicated to be effective on early return to work in patients with musculoskeletal diseases in general [20] and in low back pain patients in particular [21]. But there is no convincing effect on the single elements [22,23]. Similarly, no effect was found from ergonomic intervention alone [24,25]. It seems to be the combination of the elements that has an effect on RTW in LBP patients [2]. European guidelines for management of both acute and chronic LBP recommend screening for psychosocial factors, including fear avoidance beliefs behaviour related to both physical activity and work [26].

In Denmark, RTW coordinators are traditionally case managers at the municipal sickness benefit offices. The coordination will at the earliest begin after 8 weeks of sick listing. In a recent review, Shaw et al. [27] recommend that the RTW coordinator has a medical background (i.e. physiotherapist, occupational therapist). This might enable the coordinator to give a realistic prognosis of when the patients are ready for returning to work, full time or part time, and to make recommendations about a possible return to sub elements of their jobs. The physiotherapist is in this study chosen to be coordinator since he/she is the healthcare practitioner, who has regular contact on a weekly basis during the rehabilitation process. Additionally the physiotherapist can test the physical function during progress of treatment comparably to the work load the patient is exposed to at work.

### How does this intervention differ from earlier interventions?

Previous Danish multidisciplinary interventions on RTW in patients with LBP and other musculoskeletal disorders have not been able to demonstrate convincing results [28,29]. In contrast, multidisciplinary intervention studies from Canada and the Netherlands have demonstrated positive effect on shortening the period of sick leave in sick listed workers [30,31]. A recent Danish study testing a simple short-time intervention by an occupational physician, showed short time effect on disability and sick leave [32]. The present study differs from the earlier interventions on several crucial points:

•Earlier interventions have mainly focused on persons, who were already sick listed. In the present study, we also wish to test a preventive intervention in patients, who are working, but at risk of being sick listed due to LBP.

•The objective of the treatment in this study is not necessarily total pain relief, but managing a life with recurrent episodes of LBP in both work and leisure time. The study is made in the framework of improving or retaining the workability in LBP patients despite of their LBP.

•This is the first study, where the physiotherapist, who is currently treating the patient, also has the role of RTW-coordinator in a multidisciplinary team. The physiotherapist is the main contact for the patient during the rehabilitation process, and has the most coherent contact with the patient (1–2 times pr. week). This provides a confident and trusting contact, coherence in the rehabilitation process and a fast and systematic involvement of relevant stakeholders from the multidisciplinary team.

•The case manager from the municipal sickness benefit office is a part of the multidisciplinary team, and since she is participating in the meetings, decisions can be made without delays.

•The new cooperative relations developed in this study are based on existing organizations in the city of Copenhagen. Thus the intervention can easily be implemented in the future, if found effective.

The overall focus of the intervention will be:

•Multidisciplinary management within two weeks after inclusion.

•Close cooperation between the stakeholders at the rehabilitation centre, the local hospital, the sickness benefit office, and in particular the patients’ workplace.

•A rehabilitation plan based on a bio-psycho-social approach, with timing of the various interventions.

### Aim

The aim of *this paper* is to describe the study protocol, design, and the content of the intervention in the TIKI-project. We provide a detailed description of all the elements in the early coordinated intervention and of the criteria for the involvement the various participants in the multidisciplinary team.

The overall aim of the TIKI study is to evaluate the effect of a work oriented multidisciplinary intervention, coordinated by the treating physiotherapist, on sickness absence in LBP patients compared to usual care.

In addition we wish to develop new cooperative relations in the early multidisciplinary coordinated intervention, which involve all stakeholders around the LBP patient in a shared rehabilitation plan. Finally, we wish to identify risk factors for poor outcome from the intervention.

### Outcomes

The main outcome is sickness absence, measured as number of days off work 12 months after inclusion.

The secondary outcomes are: number of patients who have returned to work, pain, back-related disability, quality of life, and community costs.

Outcomes are measured at baseline, at the end of treatment as well as 6 months and 12 months after randomization.

## Methods/design

### Study design

A non-blinded randomized controlled trial.

### Study population

Patients with low back pain referred to treatment at the Copenhagen Back- and Rehabilitation Centre from either a general practitioner, rheumatologist or from the municipal sickness benefit office.

### Inclusion procedure

A written information sheet is sent by mail to all eligible patients, and within two days a phone call is made by a trial secretary to determine if the patient meet the inclusion criteria and is interested in participating in the study. In order to report characteristics in not eligible patients, all data on cause of exclusion is collected.

### Criteria for inclusion

Working age adults (18–65 years) with LBP for more than two weeks. The participants can be employed or unemployed, sick listed or at risk of sick listing.

### Criteria for exclusion

Comorbidity (i.e. severe consequences of cancer, cardiopulmonary diseases, mental/psychological diseases), pregnancy, difficulties in reading and writing the Danish language or application for early retirement or “occupational rehabilitation” (reassignment to another type of occupation economic subsidised, a unique Danish constellation).

### Procedure of inclusion and randomization

**Figure 1:**
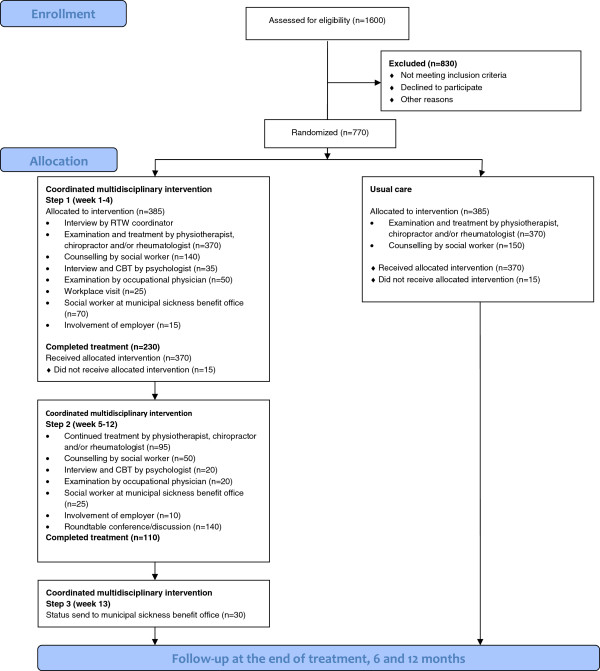
Recruitment and expected patient flow.

The patient will meet the project manager for an interview at the Back- and Rehabilitation Centre within one week after the phone call. After signing an informed consent form, all patients fill in a questionnaire (see “measurements” below). The inclusion interview and completion of questionnaires is completed before the randomisation without either patient or study mangers knowledge of group allocation. Random allocation is produced by a computerized random number generator in blocks of ten. The group allocation is handled by an independent trial secretary. The flow in the study is shown in Figure 1.

Blinding: Because of the nature of the treatments, it is not possible to blind patients and treatment team to the intervention. However; researchers and statisticians, who will obtain and assess outcomes are blinded to assignment.

### Measurements

The questionnaires cover demographic and personal data: age, sex, marital status, BMI, educational level, occupation, possible sick listing, duration, and economic relief. Work related factors: job satisfaction, working hours, self-assessed workability, and beliefs on working future. Lifestyle factors: physical activity, smoking, and alcohol consumption.

### Sickness absence

Sickness absence is measured in days from first day off work until last day of full time sickness absence. To measure the number of days off work during the 12 months follow up, we use both self-reported data and data from a national database by The Danish Ministry of Employment (the DREAM Database), where data on social transfer payments including sickness benefits are available [33].

### Pain

Pain intensity is measured on the Numerical Rating Scale, NRS [34], were the patient is asked to mark current/actual pain, worst pain in the previous two weeks and average pain in the previous two weeks. Both LBP and leg pain is registered. Duration of pain, earlier episodes of LBP and use of analgesics is also recorded.

### Disability

The Roland Morris Questionnaire, RMQ [35,36], is used to explore change in self-reported disability due to/related to LBP. The Danish language version of RMQ is a 23 item questionnaire; each item is qualified by the phrase: “because of my back pain” [36]. The items will be scored “yes” or “no”.

### Health status

For general health and quality of life the Short Form 36 questionnaire, SF-36, is used [37,38]. SF-36 is a generic tool that measure general or functional health. SF-36 generates 8 subscales and two summary scores. The 8 subscales are: physical functioning, role limitations due to physical problems, bodily pain, general health perceptions, vitality, social functioning, role-limitations due to emotional problems, and mental health. The two summary scores are the physical component summary and the mental component summary.

### Psychological distress

The Danish language version of The Symptom Checklist-90-Revised, SCL-90-R, is used to measure psychological distress [39]. The SCL-90-R is a screening tool of general psychiatric symptomatology, which consists of 90 items, divided in 10 subscales measuring: somatization, obsessive-compulsive, depression, anxiety, phobic anxiety, hostility, interpersonal sensitivity, paranoid ideation, psychoticism and an additional scale concerning sleep and appetite [40]. The 90 items are scored on a five point Likert scale indicating the degree to which the person has been distressed by the symptom in the past week.

### Fear avoidance beliefs

Fear avoidance behaviour is when a person seeks to avoid activities that cause pain. Using the Fear-Avoidance Beliefs Questionnaire (FABQ) it is possible to identify LBP patients at risk of developing fear avoidance behaviour. The FABQ [41,42] is used to assess patients' beliefs about how physical activity and work affect their low back pain. The questionnaire consists of 16 questions to be answered on a 7 point Likert scale. It is divided in two sub scales covering physical activity (FABQ-PA) and work (FABQ-W).

### Economic evaluation

To compare society costs in the intervention group compared to the usual care group cost utility analysis will be conducted. The cost utility analysis will include the 770 LBP patients in the RCT. Expenses for health care and sickness absence during the intervention period and at 12 months follow-up will be included in the analysis.

Number of treatments and consultations will be registered subsequent during the intervention. The patients will report the use of health care services during the follow-up in questionnaires. Occupational status (employed or unemployed) and data on sickness absence retrieved from a national database (the DREAM registry) will be included. Based on the SF-36 questionnaire a health status utility value is calculated for each participant, the SF6D score [43]. The SF6D utility score is used to construct quality adjusted life years (QALY) and to conduct cost-utility analysis.

### Interventions

#### Treatment in the control group (usual care)

Standard treatment at Back and Rehabilitation Centre Copenhagen consist of examination and treatment by physiotherapists, chiropractors, rheumatologists and if needed, counselling by a social worker. The examination includes a standardized mechanical evaluation and individually chosen advice on exercise. General advice is given to increase physical activity. The examination and treatment is based on a bio-psycho-social approach according to the recommendations from Back Pain Europe’s evidence based guide lines [26]. The initial examination is usually performed by a physiotherapist or a chiropractor and consists of a thorough exploration of biomechanical and neuro-dynamic conditions. The patient will be examined by a **rheumatologist** if there is a need for diagnostic examination, for example with a suspected severe LBP pathology (herniated disc, spinal stenosis, fracture, spondylolisthesis, tumours, osteoporosis etc.) or if the patient does not have the expected effect of treatment. Additionally if medication or steroid injections is needed, if there is suspicion of inflammatory diseases (i.e. rheumatoid arthritis, Mb. Bechterew), or if differential diagnostic elucidation is required (including x-ray or MRI) the rheumatologist is involved.

A social worker is employed at the rehabilitation centre to offer counselling to the patients. The social worker has no authority concerning economic issues in the actual case, but provides counselling due to a broad knowledge of the social- and sickness benefit law/legislation and has a close communication with the sickness benefit office.

#### Treatment in the intervention group

In addition to usual treatment described above, participants allocated to the intervention group will be met by a multidisciplinary approach.

### RTW coordinator

**Figure 2:**
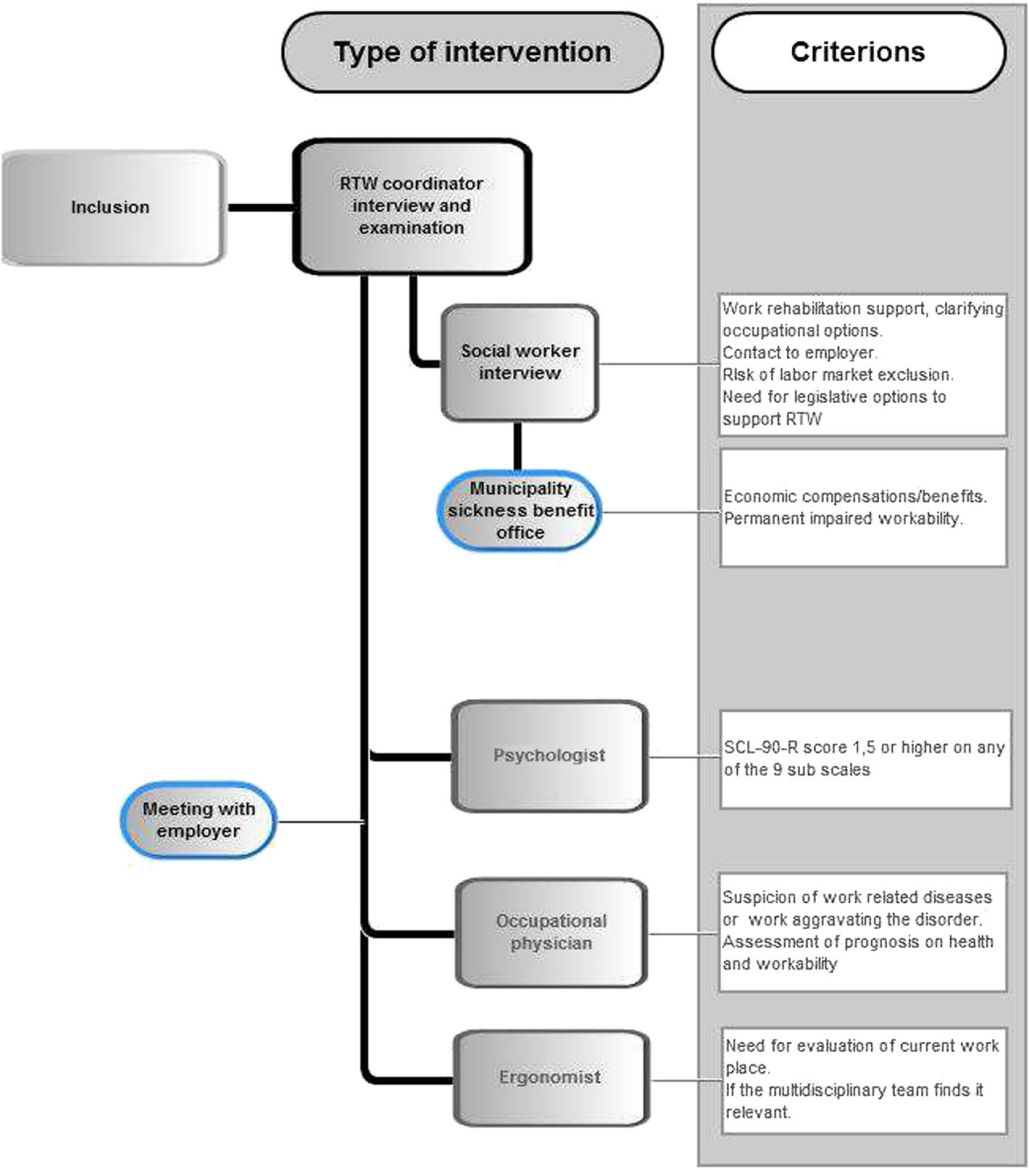
Criterions for involvement of the health professionals in the intervention group.

The patient will meet a RTW coordinator at the first visit after inclusion in the study. The RTW coordinator is a trained physiotherapist, who will assist the patient throughout the treatment. In a limited number of patients a chiropractor will be RTW coordinator. This is if the patient earlier has experienced positive effect from manipulative therapy, and is specifically referred to a chiropractor. The same physiotherapist or chiropractor is treating and advising the patient on exercises and physical activity (see description above). The role of the coordinator is to ensure timeliness of the different interventions, facilitate the communication between all stakeholders, and maintain contact with the workplace; in particular make a recording of the patients’ work abilities and the demands at the workplace [44]. The different elements in the intervention are aimed to be timed parallel; not serial to condense the intervention time as much as possible. The coordinator must ensure the timeliness of the various elements and that the whole intervention is on track (Figure 2).

#### Social worker at the back- and rehabilitation centre

The task for the social worker at the Back- and Rehabilitation Centre is to explore the potential needs for support according to the work rehabilitation, and will meet with the patient within the first week after inclusion. The social worker keeps continuous contact to the case manager at the municipal sickness benefit office.

### Case manager at the municipal sickness benefit office

The case manager at the municipal sickness benefit office holds responsibility when economic compensations and specific initiatives to ensure fast RTW or work retention are required. Since the case manager from the municipal sickness benefit office is part of the team, it is possible to make decisions about and to effectuate any initiative e.g. on economic compensations without a long processing time.

### Psychologist

If a patient scores more than 1.5 on any of the 10 sub scales in SCL-90-R he/she is offered **cognitive behavioural therapy (CBT)**. The RTW coordinator will arrange a consultation with a psychologist who will reveal social and personal strains related to LBP and work.

### Occupational physician

The RTW coordinator will arrange a consultation with an occupational physician for assessment and advice regarding the patients’ work ability:

•If work related disorders are suspected.

•If there is a suspicion, that working aggravates the disorder.

•Suggestions for job tasks that can be tolerated at the current health status.

•Assessment of the prognosis on health and workability.

### Ergonomist

The RTW coordinator will arrange a work place visit by the specialist in ergonomic evaluation for assessment of the patients’ current work place concerning:

•Exploration of the physical arrangement and organization of the work place.

•Recommendations for job modifications or ergonomic changes, temporary or permanent.

•Ergonomic counselling regarding both job and daily life.

After the work place visit, the ergonomist composes a report to the team. The recommendations for job modifications and ergonomic changes are presented for the employer and the municipal case manager, who will authorize the expenses.

### Multidisciplinary team meetings

Monthly all members of the multidisciplinary team meet to discuss and organise the rehabilitation process for new study participants and to evaluate the plans and interventions for the more complex cases. These meetings enable a close cooperation between health professionals and municipal office workers. And the processing time in cases, that require any initiative concerning economic compensations can be shortened.

### Timeframe in the intervention

#### Step 1 (0–4 weeks after inclusion)

The overall focus in the first phase of treatment is examination, identifying barriers of returning to work or risk factors for absence from work, and involvement of relevant collaborators from the team within the first two weeks. At the first consultation the patient and RTW coordinator discuss the results from the screening (psychosocial distress and work ability). The patient is examined as described above. Within the first week, the patient will meet the social worker employed at the rehabilitation centre.

#### Step 2 (4–8 weeks after inclusion)

If no significant improvement concerning pain and function is obtained, or if the patient still has not resumed work (full time, part time or with modified tasks), the patients’ situation will be discussed in the team. Supplementary examination, round table conference at the workplace, and “therapeutic return to work” can be effectuated.

#### Step 3 (8–12 weeks after inclusion)

If return to work at this stage has still not been possible, a written health status is send to the municipal sickness benefit office to initiate further intervention.

The time frame of the multidisciplinary intervention is maximally twelve weeks; however it is possible to continue training and treatment in the Back and Rehabilitation Centre for more than 12 weeks. Data on number of consultations and duration of the treatment period is successively obtained in both groups.

### Data collection

At the inclusion interview, prior to randomization and again at the last consultation the patient will fill in the previous mentioned questionnaires at the Back- and Rehabilitation Centre. The follow up questionnaires at 6 and at 12 months after inclusion will be sent to the patients by mail with stamped addressed envelopes for returning by an independent secretary with no knowledge of group assignment. The study is currently on-going and by December 1^st^ 2012 600 (out of 770) patients are recruited. It is expected that the data collection will be finished during 2014, including the 12 months follow up.

### Statistics

#### Sample size

Sample size is calculated based on the main outcome, number of days off work, assuming an average difference between groups of 20% (5 days at an average absence period of 25 days), a dispersion of 22 days (25% of a range of 90 days) and a level of statistical significance at 0.05 and a power of 0.8. These assumptions mean that 305 participants need to complete the treatment in each of the two groups. Accounting a possibly high rate of withdrawal or loss to follow-up at 20% in this population; 770 individuals need to be recruited for the study. Assessment is blinded and will follow the intention-to-treat principle, including all randomized participants who provide follow-up data. All data will be handled as described and recommended in the CONSORT statement [45].

#### Data analysis

The data analysis of differences between effect in the intervention and the control group will be undertaken according to intention-to-treat principles. Cox proportional hazard models will be used to analyse time till RTW and financial independence, by which the variation in observation time and time until RTW is considered. Analysis will be carried out to control for potential confounders (e.g. sex, age, education level, occupational status) [46].

## Discussion

### Strength and limitations of the TIKI study

The main outcome in this study is number of days off work and it is collected as register data from a national database, eliminating recall bias and allowing us to get data on this parameter from non-responders.

In LBP research investigating the effect of a multidisciplinary intervention, treatment in the control group is often a single profession approach. In this study the control group will receive an evidence based treatment from physiotherapist, chiropractor and rheumatologist (usual care). Blinding of the practitioners performing the interventions was not possible. For both interventions, however, practitioner preference bias was minimized by choosing practitioners who strongly believed in the treatments that they performed.

The present set-up and the cooperation between the different stakeholders and health professionals are conducted within the existing organisation. Patients are referred from GP to treatment at the Back- and Rehabilitation Centre as usual, but will be asked to participate in the study when the contact to the Back and Rehabilitation Centre is made. If the intervention in this study appears to be effective in reducing disability and increasing RTW, it can easily be implemented in a Danish setting in daily practice in the future. Although the potential impact and the design of the intervention will fit in the Danish context best, LBP has general interest internationally and it should be possible to extrapolate the results or at least parts of it to a setting in another society.

A number of challenges will also be met in this study; there might be some risk of medicalization of the patients by having too many health professionals focusing on their rehabilitation. However; the RTW coordinator will only involve the relevant members of the multidisciplinary team according to strictly stipulated criterions.

### Concluding remarks

Since the study is on-going and 600 of 770 patients are recruited, we have already made some preterm experiences:

Due to the actual financial crisis, the conditions at the labour market have changed and a substantial portion of the sick listed participants in the study have lost their job - either before inclusion or during the period of the intervention. This means that the amount of persons receiving a work place visit is reduced and lower than expected during the study design.

On the other hand the need for interviews by the psychologist appears larger than estimated, since a large amount of participants have a score higher than 1,5 on either of the subscales on SCL-90-R. Presumably we can transfer and modify means between posts; otherwise, if not all patients meeting the criterions for interview by a psychologist can in fact see a psychologist, this will be a potential limiting factor in the study.

The first results from the present study will be available in 2014.

### Ethical considerations

The study meets the declaration of Helsinki II, since there is no risk for the participants and it is possible to withdraw the given consent at any time.

The TIKI study is approved at the Regional Ethics Committee, Region Hovedstaden, Denmark (J.nr: H-C-2008-112) and has also been notified to and registered by the Danish Data Protection Agency. This study is registered at the ClinicalTrials.gov (Id: NCT01690234).

## Competing interests

The authors declare that they have no competing interest.

## Authors’ contributions

All the authors (AF, HL, TP and OM) took part in the design of the study. AF and TP are responsible for the daily operational aspects of the study including data collection. AF wrote the first drafts of the study protocol. All authors participated in the preparation of the manuscript and all authors read and approved the final manuscript.

## Pre-publication history

The pre-publication history for this paper can be accessed here:

http://www.biomedcentral.com/1471-2474/14/93/prepub
